# The International Review

**Published:** 1874-02

**Authors:** 


THE INTERNATIONAL REVIEW.—This splendid Review has been changed from a quarterly to a bi-monthly publication. The number for January has a rich table of contents. Our late Panic; Fires in American Cities, by Prof. Peabody; Deep-sea Exploration, by Prof. Carpenter, (London); Universal Education, by Dr. Palmer; The Prussian Church Law, by Baun Franz Von Holtzendorff; International Arbitration, by Dr. Woolsey, give forth no uncertain sound as to the quality and quantity of the contents. A. S. Barnes, 111 and 113 William street, New York. Price $5.00. Single number $1.00.

